# Detection of antimicrobial-resistant Enterobacterales in insectivorous bats from Chile

**DOI:** 10.1098/rsos.231177

**Published:** 2023-11-08

**Authors:** Zulma Esperanza Rojas-Sereno, Daniel G. Streicker, Tania Suarez-Yana, Michelle Lineros, Verónica Yung, Sylvain Godreuil, Julio A. Benavides

**Affiliations:** ^1^ Centro de Investigación para la Sustentabilidad y Doctorado en Medicina de la Conservación /Facultad Ciencias de la Vida, Universidad Andrés Bello, República 440, Santiago 8320000, Chile; ^2^ School of Biodiversity, One Health and Veterinary Medicine, University of Glasgow, Glasgow G12 8QQ, UK; ^3^ MRC-University of Glasgow Centre for Virus Research, Glasgow G61 1QH, UK; ^4^ Sección Rabia, Departamento Laboratorio Biomédico, Instituto de Salud Pública de Chile, Santiago 8320000, Chile; ^5^ Laboratoire de Bactériologie, Centre Hospitalier Universitaire de Montpellier, Montpellier 34295, France; ^6^ Laboratoire Mixte International, DRISA, IRD, Montpellier 34394, France; ^7^ MIVEGEC, IRD, CNRS, Université de Montpellier, Montpellier 34394, France

**Keywords:** *Tadarida brasiliensis*, antimicrobial resistance, *Rahnella aquatilis*, urban, extended-spectrum beta-lactamase, Latin America

## Abstract

Enterobacterales of clinical importance for humans and domestic animals are now commonly detected among wildlife worldwide. However, few studies have investigated their prevalence among bats, particularly in bat species living near humans. In this study, we assessed the occurrence of Extended-spectrum beta-lactamase-producing (ESBL) and carbapenemase-resistant (CR) Enterobacterales in rectal swabs of bats submitted to the Chilean national rabies surveillance program from 2021 to 2022. From the 307 swabs screened, 47 (15%) harboured cefotaxime-resistant Enterobacterales. Bats carrying these bacteria originated from 9 out of the 14 Chilean regions. Most positive samples were obtained from *Tadarida brasiliensis* (*n* = 42), but also *Lasiurus varius*, *L. cinereus* and *Histiotus macrotus*. No Enterobacterales were resistant to imipenem. All ESBL-Enterobacterales were confirmed as *Rahnella aquatilis* by MALDI-TOF. No other ESBL or CR Enterobacterales were detected. To our knowledge, this is the first screening of antibiotic-resistant bacteria in wild bats of Chile, showing the bat faecal carriage of *R. aquatilis* naturally resistant to cephalosporins, but also including acquired resistance to important antibiotics for public health such as amoxicillin with clavulanic acid. Our results suggest unknown selective pressures on *R. aquatilis*, but low or no carriage of ESBL or CR *Escherichia coli* and *Klebsiella* spp. Future studies should assess the zoonotic and environmental implications of *R. aquatilis*, which are likely present in the guano left by bats roosting in human infrastructures.

## 1. Introduction

Antimicrobial resistance (AMR) circulating at the human–domestic–wildlife interface is a global threat to public health, causing at least 1.27 million human deaths per year [1,2]. Extended-spectrum beta-lactamase-producing (ESBL) and carbapenemase-resistant (CR) Enterobacterales including *Escherichia coli* and *Klebsiella pneumoniae* represent the highest burden of deaths attributed to AMR in humans. Antimicrobial-resistant bacteria are carried by wildlife worldwide, and are often considered as an indicator of wildlife exposure to anthropogenic pathogen sources [2–9]. Wildlife can act as sentinels of ‘human pathogen pollution’ in natural environments, but could also become potential reservoirs of AMR to humans or domestic animals [4,7,10,11]. Bats (order Chiroptera) are considered one of the main reservoirs of human infectious diseases, because they harbour a large number of zoonotic viral pathogens, and their global distribution widely overlaps with humans [12]. However, the role of bats in the dissemination of AMR remains still poorly understood [7].

Recent studies have investigated the occurrence of AMR among bats worldwide, mostly reporting the faecal carriage of ESBL-*E. coli* [7,13–19]. For example, ESBL-*E. coli* have been reported in *Eidolon helvum*, *Megaloglossus woermanni* and *Nycteris hyspida* in Africa and *Tadarida teniotis* in Europe [18,20,21]. In Latin America, ESBL-*E. coli* have been reported in frugivorous bats including *Artibeus planirostris* and *Sturnina lilium* of Brazil, the omnivorous *Phyllostomus hastatus* in Trinidad, insectivorous bats (*Pteronotus parnelli*, *Eptesicus tadeii* and *Molossus* spp.) in Brazil and Trinidad, and the hematophagous *Desmodus rotundus* in Peru and Brazil [7,13–15,22]. ESBL and CR-*E. coli* have also been reported in the frugivorous bats *Artibeus lituratus* and *Carollia perspicillata* in Brazil [22]. However, little is known about ESBL-*E. coli* in other non-tropical regions of Latin America including Chile.

Antimicrobial-resistant bacteria found in bats are commonly hypothesized to be attributable to contamination from humans or livestock [13,19]. However, few studies have compared the prevalence of these bacteria among natural, rural and urban areas [7,18,20–22]. As such, the role of human activities and the ability of bats to spread AMR within their populations remains poorly understood. In Chile, ESBL-*E. coli* have been isolated from wild rodents, gulls, and condors [23–26], but no study has been conducted on bats. Chile hosts several insectivorous bats inhabiting urban and rural areas that are subject to the passive rabies surveillance program of the National Institute of Health [27]. Among insectivorous bats submitted to the surveillance program, *T. brasiliensis* is one of the bat species most closely associated with humans [28]. In this study, we aimed to estimate and compare the prevalence of ESBL and CR-Enterobacterales among Chilean bats from rural and urban areas.

## 2. Material and methods

We collected 307 rectal samples from dead bats that were submitted to the National Rabies Surveillance Program of the Institut of Public Health (Instituto de Salud Pública, ISP) in Chile from February 2021 to August 2022. Bats studied originated from 14 geographical regions and belonged to the genera *Tadarida*, *Histiotus*, *Lasiurus*, and *Myotis*. Morphological identification of bat individuals was performed by ISP staff, and was based on key distinctive features of each species. All bats studied here were negative for rabies according to the direct fluorescent antibody test. Samples were stored in Stuart transport media at 4°C until being screened in MacConkey selective media supplemented with antibiotics to isolate ESBL and CR Enterobacterales. Samples were also grown on a MacConkey media without antibiotics (negative control), to confirm the presence of Enterobacterales on the faecal sample. Screening was made by direct incubation at 37°C for 24 h on MacConkey media supplemented with cefotaxime sodium salt (CTX) at 2 mg/ml for ESBL-Enterobacterales and imipenem (IM) at 4 mg/ml for CR-Enterobacterales screening, following previously published methods [7,13]. Samples presenting bacterial growth were purified in the same medium to confirm growth and stored at −80°C. Bacterial species confirmation was performed on 32 randomly chosen ESBL isolates among a total of isolates with similar morphology and antibiotic-resistant phenotypic profile, using the matrix-assisted laser desorption ionization-time of flight (MALDI-TOF) mass spectrometry using the MALDI Biotyper database DB 8326 MSP (Bruker Daltonics, Bremen, Germany) in the Laboratory of Microbiology of the Hospital Arnaud de Villeneuve in Montpellier (France). Susceptibility tests were performed using the disc diffusion method on Müller-Hinton agar following the European Committee on Antimicrobial Susceptibility Testing (EUCAST) guidelines (Version 7.1, 2017). Twenty-seven antibiotics were analysed including penicillin (ampicillin, amoxicillin, ticarcillin, piperacillin and temocillin), penicillin with β-lactamase inhibitors (amoxicillin-clavulanic acid), antipseudomonal penicillin with β-lactamase inhibitors (ticarcillin-clavulanic acid and piperacillin-tazobactam), non-extended spectrum cephalosporins (cephalexin), extended-spectrum cephalosporins (cefotaxime, ceftazidime, cefepime and cefpodoxime), cephamycin (cefoxitin), carbapenems (imipenem, ertapenem, meropenem), monobactams (aztreonam), quinolones (ofloxacin, ciprofloxacin and levofloxacin), aminoglycosides (gentamicin, tobramycin and amikacin), folate pathway inhibitors (trimethoprim-sulfamethoxazole), phenicol (chloramphenicol) and phosphonic acids (fosfomycin). We divided antibiotics into the ‘categories' defined in Magiorakos *et al*. [29].

To evaluate the difference in occurrence and prevalence between bats inhabiting rural and urban areas, we conducted two complementary statistical tests. First, each municipality was assigned as rural or urban following the Chilean ‘Comisión Interministerial de Ciudad, Vivienda y Territorio-COMICIVY'T' (https://www.masvidarural.gob.cl/ruralidad-en-chile/). A municipality was assigned as urban if more than 75% of its population were living in human densities higher than 150 inhabitants by km^2^. A Chi-square test was then used to test the overall difference in prevalence between samples originated from urban or rural municipalities using the prop.test function in R, or a Fisher's exact-test if the number of samples was small (fisher.test function). We also used a continuous variable of population density to evaluate the effect of urban areas by conducting a general linear mixed-effect model with binomial residual distribution with the glmer function [30]. The model included the dependent variable ‘presence/absence of AMR’, and year, the municipality's population density (log-transformed to increase normality and avoid outliers) or total population (square-root transformation) as independent variables (each tested in a separated model given their correlation), and the municipality within region as random effects. Chile was divided by regions and each municipality was assigned to a region using the shapefile from Biblioteca Del Congreso Nacional de Chile (https://www.bcn.cl/siit/mapas_vectoriales/index_html). The human population size and density per municipality were obtained from the Comisión Interministerial de Ciudad, Vivienda y Territorio-COMICIVYT. Since bats were collected on private property, no GPS coordinates were available in order to comply with the confidential agreement established with the ISP.

## 3. Results

**Figure 1.**
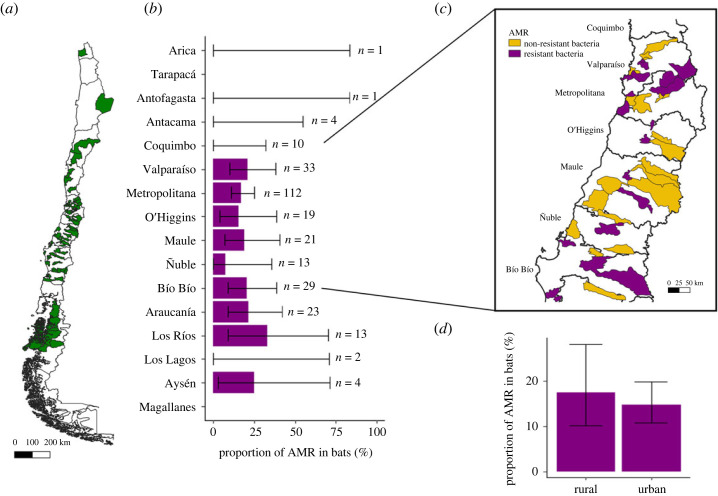
Spatial distribution of antimicrobial-resistant Enterobacterales among insectivorous bats from Chile: (*a*). Map of Chile including sampled municipalities (*b*). Bar plot represents the proportion of AMR Enterobacterales in insectivorous bats per region. The total number of individual bats sampled per region (n) is shown at the top of each confidence interval bar (estimated using the fisher.test function in R). (*c*) Zoom map of central Chile showing municipalities with bat samples that harboured antimicrobial resistant bacteria (purple) or not (yellow). (*d*). Bar plot comparing the proportion of AMR between rural and urban municipalities. A municipality was assigned as urban if more than 75% of its population was living in densities higher than 150 inhabitants per km^2^.

**Figure 2.**
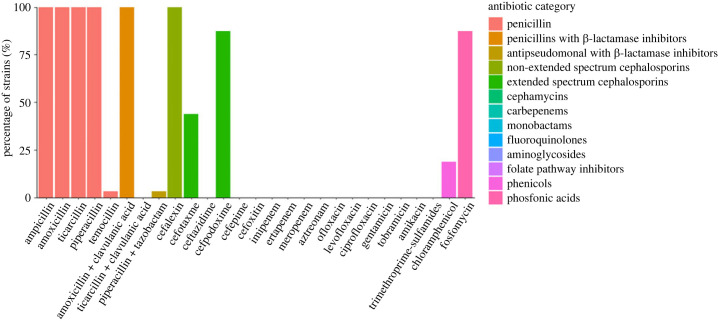
Antimicrobial resistance phenotypic profiles of ESBL-*Rahnella aquatilis* among insectivorous bats from Chile (*n* = 32). Bars represent the percentage of strains resistant to each of the 27 antibiotics tested. Colours illustrate different antibiotic categories defined by Magiorakos *et al.* [29].

From 307 faecal samples collected out of 14 Chilean regions (*n* = 238 from urban municipalities, *n* = 69 from rural municipalities, figure 1*a*), 47 bats (15%) carried CTX-resistant Enterobacterales, including 42 individuals of *Tadarida brasiliensis* (15% of AMR prevalence, 42 out of 272 *T. brasiliensis* individuals), defined as at least one isolate of CTX-resistant Enterobacterales. Two individuals of *Lasiurus varius* and one individual of *Histiotus macrotus* and *L. cinereus* also carried these bacteria (table 1). No lactose-positive Enterobacterales were resistant to imipenem. AMR-positive samples were obtained from 9 out of the 14 (64.3%) Chilean regions sampled. There was no significant difference in the prevalence between regions (Fisher's exact test: *p*-value = 0.07, figure 1*b*). AMR prevalence in urban municipalities was 11% (35 out of 307) and 17% (12 out of 69) in rural municipalities, with no significant difference between them (Chi-squared test: chi = 0.13, *p*-value = 0.72, figure 1*c*). Neither municipality's human population size (GLMM, AIC = 249, Estimate = −1.38 × 10^−3^, *p*-value = 0.16) or population density (GLMM, AIC = 251, Estimate = 0.01 × 10^−5^, *p*-value = 0.87) was significantly correlated to the presence/absence of AMR in bats. All 32 ESBL-Enterobacterales isolates (out of 47 isolates having similar morphology and antibiotic-resistant profile) were identified as *Rahnella aquatilis*. No ESBL-*E. coli* or *Klebsiella* sp. were detected. *Rahnella aquatilis* isolates were resistant to seven antimicrobial categories; 65% were resistant to five antimicrobial categories, followed by six (19%) and four (16%) categories. Resistance to antimicrobial categories included β-lactams and cephalosporins (100% to penicillin and non-extended β-lactams cephalosporins; 60% to extended-spectrum-beta-lactamases), penicillin with β-lactamase inhibitors (68% to amoxicillin with clavulanic acid), phosphonic acid (60%) and chloramphenicol (13%) (figure 2). One isolate was resistant to piperacillin with tazobactam.

**Table 1.** Prevalence of cefotaxime (CTX)-resistant Enterobacterales among insectivorous bats of Chile.

bat genera		bat species			CTX-resistant	
name	total	species	total	%	number	prevalence
*Tadarida*	272	*T. brasilensis*	272	88.3	42	13.6
*Histiotus*	26	*H. macrotus*	24	7.8	1	0.3
		*H. montanus*	1	0.3	0	0.0
		*Histiotus* sp.	1	0.3	0	0.0
*Lasiurus*	6	*L. cinerus*	1	0.3	1	0.3
		*L. varius*	4	1.3	2	0.6
		*L. villosissimus*	1	0.3	0	0.0
*Myotis*	3	*M. atacamensis*	1	0.3	0	0.0
		*M. chiloensis*	2	0.6	0	0.0
unknown	1	-	1	0.3	1	0.3
**total**			308		47	
**%**					15.3	

## 4. Discussion

The circulation of AMR in bats remains poorly understood, but recent reports have detected the presence of AMR in several bat species living in close proximity to humans and domestic animals [7,13,18]. In this study, we found cephalosporin-resistant *R. aquatilis* in three of the four genera of bats collected, mostly belonging to *T. brasiliensis,* one of the bat species with the closest association with humans in Chile and Latin America [28]. However, the lack of isolation of ESBL-*E. coli* or *Klebsiella* suggests a low or non-existent circulation of these bacteria among insectivorous bats of Chile living in proximity to humans.

We found no evidence of cephalosporin-resistant *E. coli* or *Klebsiella* spp. in bats from Chile, although these bacteria have been previously detected in bats worldwide but at a low prevalence [7,14,15,17,18,20,21]. We hypothesize that the absence or very low incidence of AMR in these bacterial species indicates a relatively low exposure with contamination sources from human or domestic animal origins in Chile, despite spatial overlapping of bats with anthropogenic landscapes. In contrast, the common vampire bat *D. rotundus* in Peru hosted ESBL-*E. coli*, potentially generated from close contact of this bat species with livestock during blood feeding [14,17,31]. Alternatively, the collection of rectal swabs from dead bats could have reduced the viability and isolation of Enterobacterales such as *E. coli*, although previous studies have found these bacteria in dead wild animals [20]. Moreover, we used the same screening method as our previous study reporting ESBL-*E. coli* in vampire bats [7], which should limit methodological biases in prevalence estimations. Therefore, our findings suggest that insectivorous bats in Chile are not common carriers of AMR, similar to the <5% prevalence of ESBL-*E. coli* and no CR found among wild mice, rabbits and foxes in central Chile [23]. Future longitudinal studies could assess if AMR prevalence evolves over time.

Although we did not find ESBL-*E. coli* and *Klebsiella* spp., we detected ESBL-*R. aquatilis* in bats from eight regions of Chile. *Rahnella aquatilis* has been previously isolated in bats from the Netherlands and Slovakia [32,33] but, to our knowledge, no report of this bacteria exists in bats of the Americas. Gerbáčová *et al*. [33] reported that *R. aquatilis* is a predominant bacterium in the insectivorous bat microbiome. This species has also been isolated from plants, water samples, and clinical samples of immunocompromised patients with bacteremia [34–36]. Thus, it can be an opportunistic bacterium causing pathogenicity in humans, and potentially in other species such as bats [32]. The isolated *R. aquatilis* from this study showed similar AMR phenotypic profiles to previous studies, with natural resistance to several families of antimicrobials such as penicillin, extended cephalosporin, and phosphonic acids [34]. We also found a few isolates of *R. aquatilis* resistant to chloramphenicol, and all isolates were resistant to amoxicillin in combination with clavulanic acid. However, resistance to ESBL in combination with clavulanic acid and resistance to chloramphenicol are uncommon in *R. aquatilis* and can be acquired [34–37], suggesting potential unknown selective pressures driving AMR to these antibiotics in the environment of Chilean bats. Future studies using whole genome sequencing could elucidate the molecular mechanisms behind the observed patterns of phenotypic resistance, and identify potential drivers for the selection of AMR in these bats.

We found no significant difference in the prevalence of ESBL-resistant Enterobacterales between urban and rural municipalities. Contrary to our expectations and despite a lack of significance, the observed tendency favoured a potentially higher prevalence in rural municipalities. Given logistical challenges and more limited surveillance in regions far away from the capital city, the number of bats submitted to the ISP surveillance program is usually higher close to urban centres and central Chile, especially for *T. brasiliensis*. Thus, comparing the difference between both environments would likely require a particular sampling effort to increase the number of bats analysed from rural municipalities. Additionally, field sample collection of live bats is required to confirm whether the observed pattern detected among dead bats of the surveillance program reflects the circulation of AMR among healthy wild bats. Finally, given our inability to obtain GPS coordinates for the location of the surveyed bats, a more refined definition of an urban/rural area where bats were found (e.g. households) was not possible. Thus, our definition of a bat as collected on an ‘urban’ or ‘rural’ area could be poorly resolved for municipalities with high spatial internal heterogeneity, but this will also depend on the extent of bat foraging within a municipality, which could expose them to most of this heterogeneity.

## 5. Conclusion

To our knowledge, this is the first screening of antibiotic-resistant bacteria in bats from Chile. We identified the faecal carriage of ESBL-*R. aquatilis* in bats, but the absence of faecal carriage of ESBL or CR-*E. coli*. Although *R. aquatilis* has been reported in bats and is naturally resistant to certain antimicrobials, resistance to β-lactamase inhibitors and chloramphenicol suggests potential mechanisms selecting for AMR on these urban and rural bats. Future studies should assess the zoonotic and public health implications of bacteria such as *R. aquatilis*, which could be largely present in the guano left by these bats in the environment.

## Data Availability

Rural or urban landscape, total population number and population density per municipality: Chilean ‘Comisión Interministerial de Ciudad, Vivienda y Territorio-COMICIVY'T (https://www.masvidarural.gob.cl/ruralidad-en-chile/). Region shapefile: Biblioteca Del Congreso Nacional de Chile (https://www.bcn.cl/siit/mapas_vectoriales/index_html). Bat information was obtained from the National Rabies surveillance program of the Instituto de Salud Pública (ISP) in Chile. Data can be publicly requested directly from ISP.
